# Identification of key miRNAs and target genes in extracellular vesicles derived from low-intensity pulsed ultrasound-treated stem cells

**DOI:** 10.3389/fgene.2024.1407671

**Published:** 2025-01-15

**Authors:** Xin Yin, Jialian Yi, Fugang Mao, Qisheng Tang, Xinyu Zhang, Xiaoyu Yang, Hongqing Xie, Linping Wang, Shuifen Sun, Xin Yu, Jie Liu, Lihong Jiang

**Affiliations:** ^1^ Faculty of Life Science and Technology, Kunming University of Science and Technology, Kunming, China; ^2^ The Affiliated Hospital of Kunming University of Science and Technology, Kunming, China; ^3^ The First People’s Hospital of Yunnan Province, Kunming, China

**Keywords:** stem cells, extracellular vesicles (EVs), low-intensity pulsed ultrasound (LIPUS), microRNAs, bioinformatics, membrane vesicles

## Abstract

**Objectives:**

This study aimed to investigate the impact of low-intensity pulsed ultrasound (LIPUS) treatment on the miRNA and mRNA profiles of stem cell-derived extracellular vesicles (EVs). Specifically, it sought to identify key miRNAs and their target mRNAs associated with enhanced therapeutic efficacy in LIPUS-treated stem cell-derived EVs.

**Methods:**

Utilizing miRNA deep-sequencing data from the Gene Expression Omnibus database, differential gene analysis was performed. MiRNA-mRNA target analysis, functional and pathway enrichment analysis, protein-protein interaction network construction, and hub gene identification were conducted. Validation of differentially expressed miRNAs was performed via RT-qPCR in human umbilical cord mesenchymal stem cells (hUC-MSCs) treated with LIPUS.

**Results:**

Ten differentially expressed miRNAs were identified, with six upregulated and four downregulated miRNAs in LIPUS-treated stem cell-derived EVs. Functional enrichment analysis revealed involvement in biological processes such as regulation of metabolic processes, cellular component organization, and response to stress, as well as signaling pathways like cell cycle, MAPK signaling, and Hippo signaling. Protein-protein interaction network analysis identified key hub genes including MYC, GAPDH, HSP90AA1, EP300, JUN, PTEN, DAC1, STAT3, HSPA8, and HIF1A associated with LIPUS treatment. RT-qPCR validation confirmed differential expression of selected miRNAs (hsa-miR-933, hsa-miR-3943, hsa-miR-4633-5p, hsa-miR-592, hsa-miR-659-5p, hsa-miR-4766-3p) in LIPUS-treated hUC-MSCs.

**Conclusion:**

This study sheds light on the potential therapeutic mechanisms underlying LIPUS-treated stem cell-derived EVs. The identified differentially expressed miRNAs and their potential target mRNAs offer valuable insights into the biological processes influenced by LIPUS treatment. While further investigation is necessary to validate their roles as therapeutic targets, this study lays the groundwork for future research on optimizing SC-EV therapy with LIPUS preconditioning.

## 1 Introduction

Stem cells (SCs) comprise undifferentiated cells possessing distinctive abilities for self-renewal and tissue regeneration, they represent a remarkable therapeutic potential and versatile applications in regenerative medicine. While SCs have shown beneficial effects in various treatments, there are some potential risks of their therapeutic use, such as tumor formation or promotion, immunological rejection and ethical concerns (Herberts et al., 2011; Stoddard-Bennett and Pera, 2020; Zhuang et al., 2021; Baranovskii et al., 2022). Recent studies have shifted the paradigm of cell-based therapy, suggesting that cell-mediated paracrine signaling, particularly through extracellular vesicles (EVs), plays a more significant role than direct cellular integration (Wernly et al., 2019; Lima Correa et al., 2021). SCs, due to their inherent regenerative potential and robust EV production capacity, emerge as ideal candidates for therapeutic EV delivery (Öztürk et al., 2021; Li et al., 2022; Tan et al., 2024).

In recent decades, bioinspired membrane vesicles, including naturally released EVs, *in vitro* self-organized cellular-membrane-derived vesicles (An et al., 2023), isolated cell-bound membrane vesicles, and others (Sun et al., 2023), have been rapidly developed as drug delivery systems. EVs are nanoscale to micron-sized membranous structures (30–1,000 nm) secreted by virtually all cell types. While traditionally classified into exosomes, microvesicles, and apoptotic bodies based on biogenesis and size, current technical limitations hinder precise differentiation (Kuriyama et al., 2021; Lötvall et al., 2014; Chuanjiang et al., 2018; Théry et al., 2018; Jeppesen et al., 2019). Therefore, the broader term “extracellular vesicles” is preferred for general characterization. EVs hold significant therapeutic potential due to their ability to deliver a diverse cargo of bioactive molecules. This cargo includes non-coding RNAs, messenger RNAs (mRNAs), proteins, and even organelles like mitochondria (Valadi et al., 2007; Abreu et al., 2020; Yue et al., 2020; Ikeda et al., 2021). The specific contents of EVs can trigger a variety of therapeutic effects, including immunomodulation, tissue regeneration, and inflammation inhibition (Cai et al., 2020; Rajool Dezfuly et al., 2021). Notably, microRNAs (miRNAs) represent a crucial component of this therapeutic arsenal (Hu et al., 2012; Kou et al., 2022).

Ultrasound referring sound waves with frequencies exceeding 20,000 Hz. Though primarily used for diagnostic imaging, medical ultrasound has seen clinical treatment applications since the 1950s (Miller et al., 2012). Therapeutic ultrasound employing higher pressures and intensities than its diagnostic counterpart, exerts mechanical stress on cells and tissues, triggering specific biological responses. Low-intensity pulsed ultrasound (LIPUS) has recently garnered considerable attention in the realm of ultrasound therapy. Multiple studies have established the modulatory role of LIPUS on EVs secretion and their subsequent therapeutic efficacy. Zeng et al. unveiled an inverse relationship between LIPUS intensity and EVs production in lung cancer cells, with lower intensities stimulating greater EVs release. In contrast, higher intensities exerted an inhibitory effect (Zeng et al., 2019). Deng et al. observed enhanced therapeutic potential in Alzheimer’s disease models treated with EVs derived from LIPUS-irradiated astrocytes compared to controls (Deng et al., 2021). Liao et al. demonstrated that LIPUS irradiation empowered bone marrow mesenchymal stem cells to secrete EVs with amplified cartilage regeneration capabilities (Liao et al., 2021). Similarly, Li et al. reported superior anti-inflammatory properties in endothelial cells treated with EVs released by LIPUS-exposed dendritic cells (Li et al., 2019). While the therapeutic potential of stem cells-derived EVs (SC-EVs) is well recognized, the mechanisms by LIPUS enhance the therapeutic efficacy of SC-EVs remain to be elucidated.

In this study, we aimed to identify miRNAs and mRNAs that play key therapeutic roles in LIPUS treated SC-EVs. To that end, we retrieved miRNA profile of EVs derived from control- and LIPUS-induced SCs. We identified miRNAs that are differentially expressed in LIPUS-induced SCs. Our study may be helpful for elucidating the mechanisms of enhanced therapeutic capacity from ultrasound stimulated SC-EVs.

## 2 Materials and methods

### 2.1 Data acquisition

The miRNA deep-sequencing data GSE188347 was obtained from the Gene Expression Omnibus database. The miRNA expression profile was generated using the GPL16791 Illumina HiSeq 2,500 platform (*Homo sapiens*). The dataset comprises EVs isolated from apical papilla stem cells (control group) and LIPUS-treated apical papilla (90 mW/cm^2^, 0.5 h; LIPUS group), with three samples each group.

### 2.2 Differential gene analysis

The R packages limma and ggplot2 were used to analyze the differences between the two groups. The miRNAs meeting the criteria *p* < 0.05 and | log2 FC| > 1.5 were identified as differentially expressed miRNAs (DEmiRNAs). Volcano plot and heatmap were generated to visualize DEmiRNAs profile. Principal component analysis (PCA) for differential gene expression was performed by the PCA online tool (Omicshar, https://www.omicshare.com).

### 2.3 MiRNA-mRNA targets analysis

The R package multiMiR was used to identify all validated target genes of the DEmiRNAs. Overlapping results from two online databases, miRTarBase (https://mirtarbase.cuhk.edu.cn) and TarBase (http://microrna.gr/tarbase), were used to filter the DEmiRNA target genes. Subsequently, the miRNA-mRNA interaction networks were extracted and visualized using Cytoscape software.

### 2.4 Functional and pathway enrichment analysis

Functional enrichment analyses for the DEmiRNA target genes were performed using the online tool OmicShare (https://www.omicshare.com). The analyses encompassed Kyoto Encyclopedia of Genes and Genomes (KEGG) pathway enrichment analysis and Gene Ontology (GO) analysis, which included terms related to biological process (BP), cellular component (CC), and molecular function (MF).

### 2.5 Protein-protein interaction network construction and hub gene identification

Validated target genes of DEmiRNAs were uploaded to the STRING database (https://string-db.org/) to predict protein-protein interaction (PPI) networks. Each node in the network represents a target gene, while edges connecting nodes indicate predicted interactions, with edge color reflecting interaction strength. Hub genes, critical players in the network, were identified using the degree cutoff criterion calculated by cytoHubba within Cytoscape software (version 3.10.1). Cytoscape was then used to visualize the resulting network and highlight these hub genes and their interactions.

### 2.6 Cell culture and LIPUS treatment

The human umbilical cord mesenchymal stem cells (hUC-MSCs) employed in this study were generously supplied by the Regenerative Medicine Research Center of Yunnan First People’s Hospital (Kunming, China). These cells were cultured in a humidified atmosphere containing 5% CO_2_ at 37°C, with an exosome-free fetal bovine serum (FBS) medium. Based on the assigned groups, the cells were subjected to either LIPUS stimulation (LIPUS group) or were left untreated with LIPUS irradiation (control group). The experimental device used was a LIPUS therapy instrument (WED-100, Well. D Medical Electronics Co., China), operating at a frequency of 1 MHz. Cells were placed in a humidified incubator and treated with LIPUS at a dose of 500 mW/cm^2^ for 10 min, with the ultrasound probe positioned 1 cm above the cell monolayer. The determination of this dose was based on our previous studies demonstrating enhanced therapeutic effects of this dose in SC-EVs. The supernatants of the cells were collected, and SC-EVs were extracted via the process of differential ultracentrifugation (Witwer et al., 2013). Briefly, cell culture supernatants were collected and subjected to sequential centrifugation steps: 300 g for 10 min to remove cells, 2,000 g for 20 min to remove debris, and 100,000 g for 90 min to pellet the EVs. The EV pellets were then washed with PBS and centrifuged again at 100,000 g for 90 min.

### 2.7 RT-qPCR analysis

The expression levels of miRNAs in SC-EVs were quantified using RT-qPCR. Total RNA, including miRNAs, was extracted from the EVs using the TRIzol reagent (Invitrogen) according to the manufacturer’s instructions. The extracted RNA was then quantified and quality-checked using a NanoDrop spectrophotometer (Thermo Scientific) to ensure its integrity and suitability before proceeding with cDNA synthesis. cDNA synthesis was performed using a miRNA first-strand cDNA synthesis kit (Sangon Biotech, China) with a universal miRNA reverse primer (5′-GTG​CAG​GGT​CCG​AGG​T-3′). miRNA-specific forward primers, as listed in Table 1, were designed and supplied by Sangon Biotech. Subsequently, RT-qPCR analysis was conducted using a SYBR Green miRNA qPCR kit (Sangon Biotech, China). Relative miRNA expression levels were calculated using the 2^^−ΔΔCt^ method, normalized to U6 small nuclear RNA (U6 snRNA) as an internal control. The primer sequences for U6 snRNA were as follows: forward primer (5′-CTC​GCT​TCG​GCA​GCA​CA-3′) and reverse primer (5′-AAC​GCT​TCA​CGA​ATT​TGC​GT-3′).

**TABLE 1:** The DE-miRNAs forward primers.

Symbol	Forward primers (5′to 3′)
hsa-miR-29b-2-5p	CTG​GTT​TCA​CAT​GGT​GGC​TTA​G
hsa-miR-3201	CGC​CGC​CAG​GGA​TAT​GAA​GAA​AAA​T
hsa-miR-935	CCA​GTT​ACC​GCT​TCC​GCT​AC
hsa-miR-933	ATA​TGT​GCG​CAG​GGA​GAC​CTC​T
hsa-miR-3943	TAG​CCC​CCA​GGC​TTC​ACT​TG
hsa-miR-4633–5p	ATA​TGC​CTG​GCT​AGC​TCC​TC
hsa-miR-592	CGC​TTG​TGT​CAA​TAT​GCG​ATG​ATG​T
hsa-miR-659–5p	AGG​ACC​TTC​CCT​GAA​CCA​AGG​A
hsa-miR-3649	GCA​GGG​ACC​TGA​GTG​TCT​AAG
hsa-miR-4766–3p	CGC​CGA​TAG​CAA​TTG​CTC​TTT​TGG​AA

### 2.8 Statistical analyses

Quantitative data were analyzed using GraphPad Prism (version 8.3.0). The choice of statistical test depended on the distribution of the data. Student’s t-test was used to compare mean values between the LIPUS group and the control group when the data met the assumptions of normality and homogeneity of variance. When these assumptions were not satisfied, the Mann–Whitney *U*-test was applied as a non-parametric alternative. The specific application of the Mann–Whitney *U*-test included comparisons where data distributions were significantly skewed or where variances were unequal, as determined through preliminary Shapiro-Wilk and Levene’s tests, respectively. A significance level of *p* < 0.05 was considered indicative of statistical significance.

## 3 Results

### 3.1 Identification of DEmiRNAs

**FIGURE 1:**
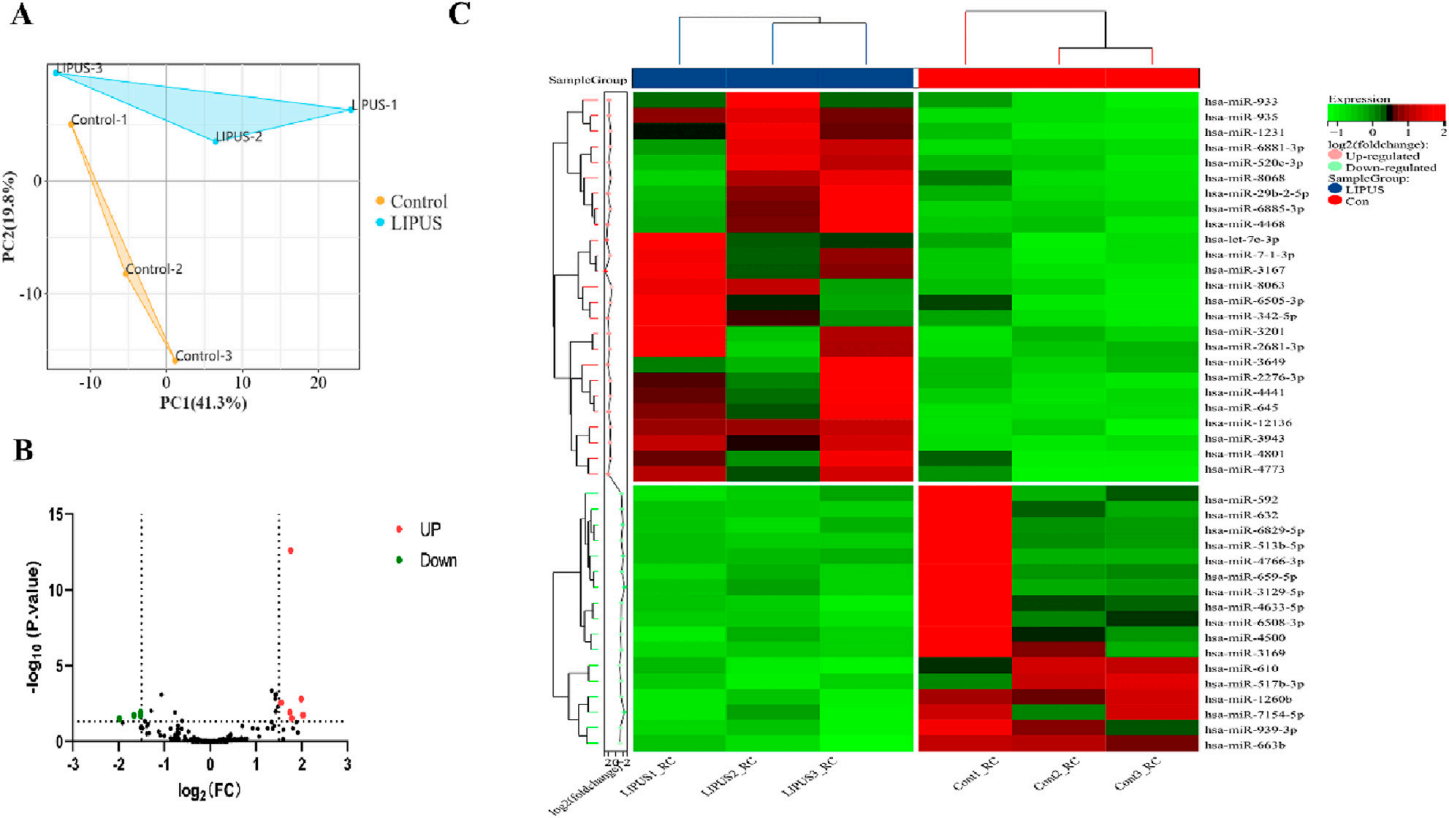
Identification of DEmiRNAs. **(A)** The principal component analysis (PCA) plot of samples in GSE188347. **(B)** Volcano plot differential gene analysis of GSE188347. **(C)** Heat map analysis of differential miRNAs.

PCA analysis distinctly segregated the LIPUS and control groups in the GSE188347 profile (Figure 1A). Utilizing the filtering criterion described above, we identified 10 DEmiRNAs, comprising six upregulated miRNAs: hsa-miR-3649, hsa-miR-29b-2-5p, hsa-miR-3201, hsa-miR-935, hsa-miR-933, hsa-miR-3943 and 4 downregulated miRNAs: hsa-miR-4633-5p, hsa-miR-592, hsa-miR-659-5p, hsa-miR-4766-3p. These DEmiRNAs were detailed in Table 2. The distribution of differential miRNA expressions between the LIPUS and control groups was visually depicted by the volcano map correlating -log10 (P-value) and log2 (FC) (Figure 1B). Additionally, a heatmap was constructed to illustrate the distinctions between the LIPUS and control groups (Figure 1C).

**TABLE 2:** The DE-miRNAs.

Symbol	FDR	LogFC	Up/Down
hsa-miR-29b-2-5p	0.001567756	1.989365949	Up
hsa-miR-3201	0.030150456	1.781029213	Up
hsa-miR-935	2.60E-13	1.758508974	Up
hsa-miR-933	0.011560008	1.736815996	Up
hsa-miR-3943	0.002766305	1.547536361	Up
hsa-miR-4633–5p	0.011560008	−1.521726729	Down
hsa-miR-592	0.019867264	−1.527456609	Down
hsa-miR-659–5p	0.019867264	−1.666177116	Down
hsa-miR-3649	0.018581031	2.028616627	Up
hsa-miR-4766–3p	0.032002154	−1.982641196	Down

Abbreviations: FDR, false discovery rate; LogFC , Log2 fold-change.

### 3.2 MiRNA-mRNA targets analysis

**FIGURE 2:**
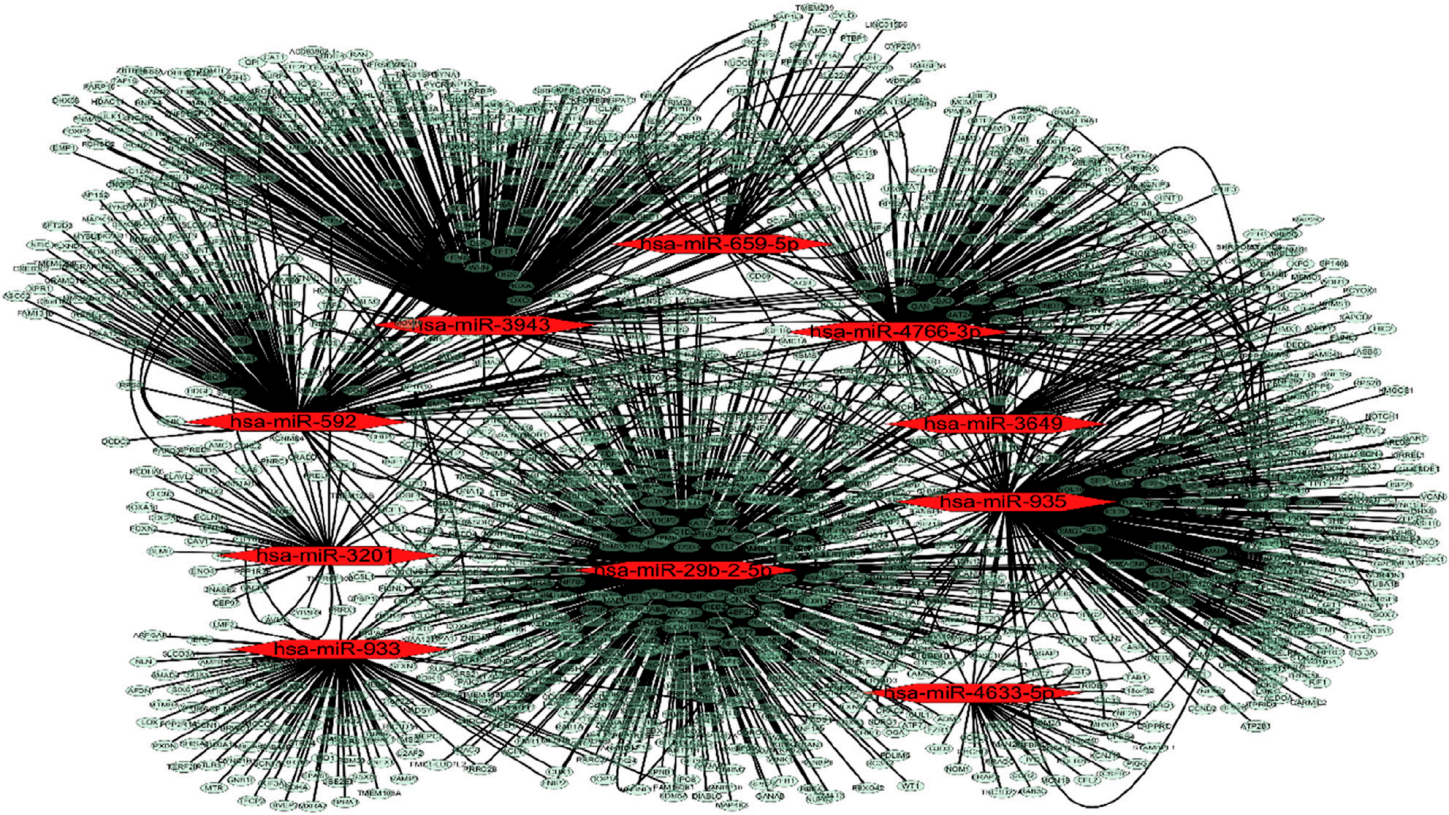
MiRNA-mRNA targets analysis. MiRNA-mRNA interactions network. The red dot represents miRNAs and the pale dot represents target mRNAs.

A miRNA-mRNA regulatory network encompassing 10 miRNAs and 1,597 mRNAs was constructed. The 1,597 mRNAs were derived from the overlapping results of validated target genes from two databases, miRTarBase and TarBase. The network depicting miRNA-mRNA interactions was visualized using Cytoscape (Figure 2).

### 3.3 Function and pathway analysis of target mRNAs

**FIGURE 3:**
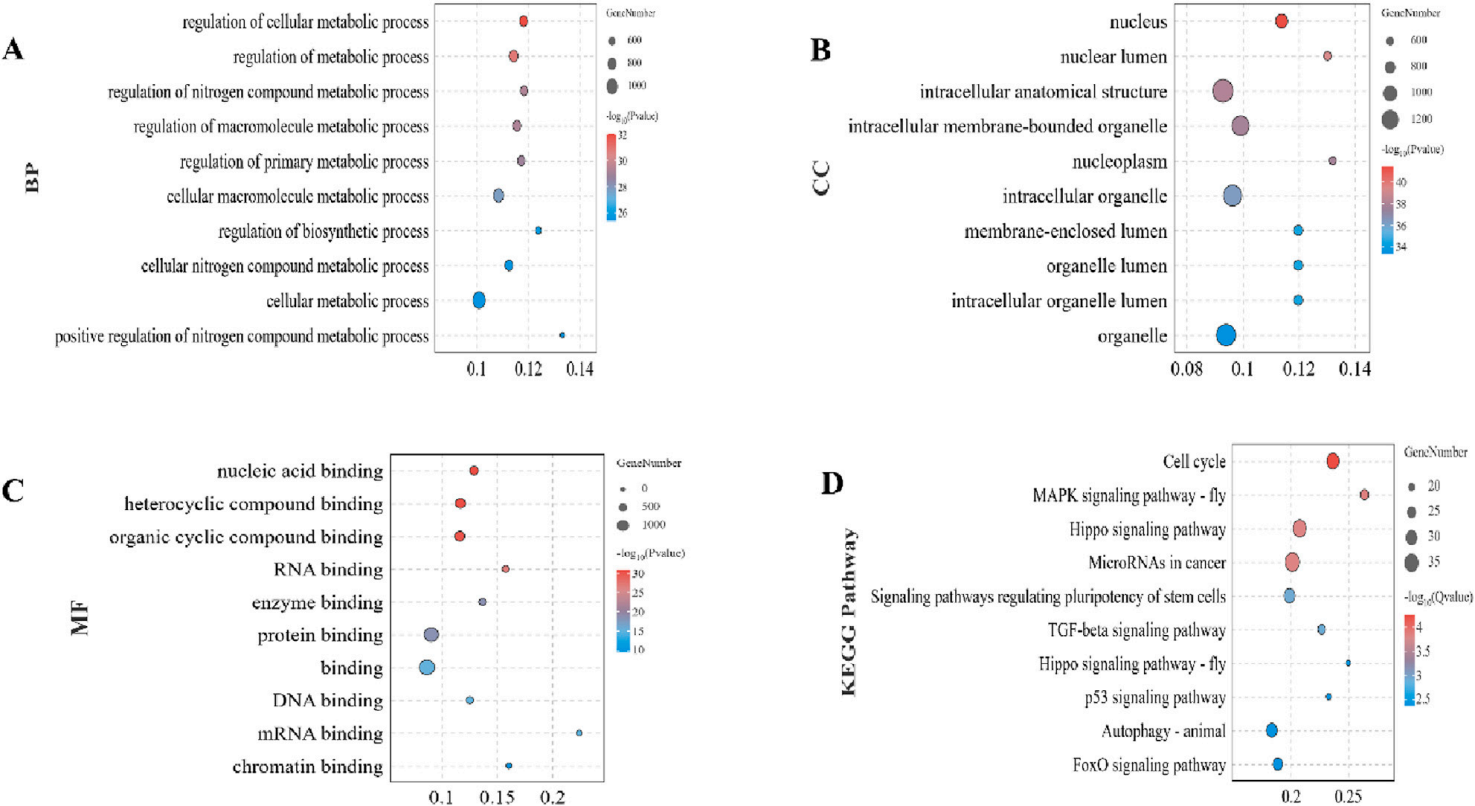
Functional and pathway enrichment analysis of target mRNAs. **(A)** Top 10 significantly enriched Biological Process (BP) terms associated with differentially expressed miRNAs. **(B)** Top 10 Cellular Component (CC) terms. **(C)** Top 10 Molecular Function (MF) terms. **(D)** Significantly enriched KEGG pathways. Enrichment analysis was performed using the OmicShare tools with significance criteria set at *p* < 0.05.

To elucidate the potential biological functions of the identified target mRNAs, we performed GO and KEGG enrichment analyses. Among the BP terms, the top 10 significantly enriched categories were associated with the regulation of metabolic processes, focusing on cellular, macromolecular, and nitrogen compound metabolism (Figure 3A). CC terms primarily pointed towards nuclear and intracellular compartments, including the nucleus, nucleoplasm, and various membrane-bound organelles (Figure 3B). MF analysis revealed enrichment in diverse binding functions, particularly involving nucleic acids, proteins, and RNA (Figure 3C). Analysis of KEGG pathways identified significantly enriched pathways, including cell cycle, MAPK signaling, Hippo signaling, microRNA involvement in cancer, pluripotency regulation, and various signaling pathways related to growth and development (Figure 3D).

### 3.4 PPI network construction and hub genes analysis

**FIGURE 4:**
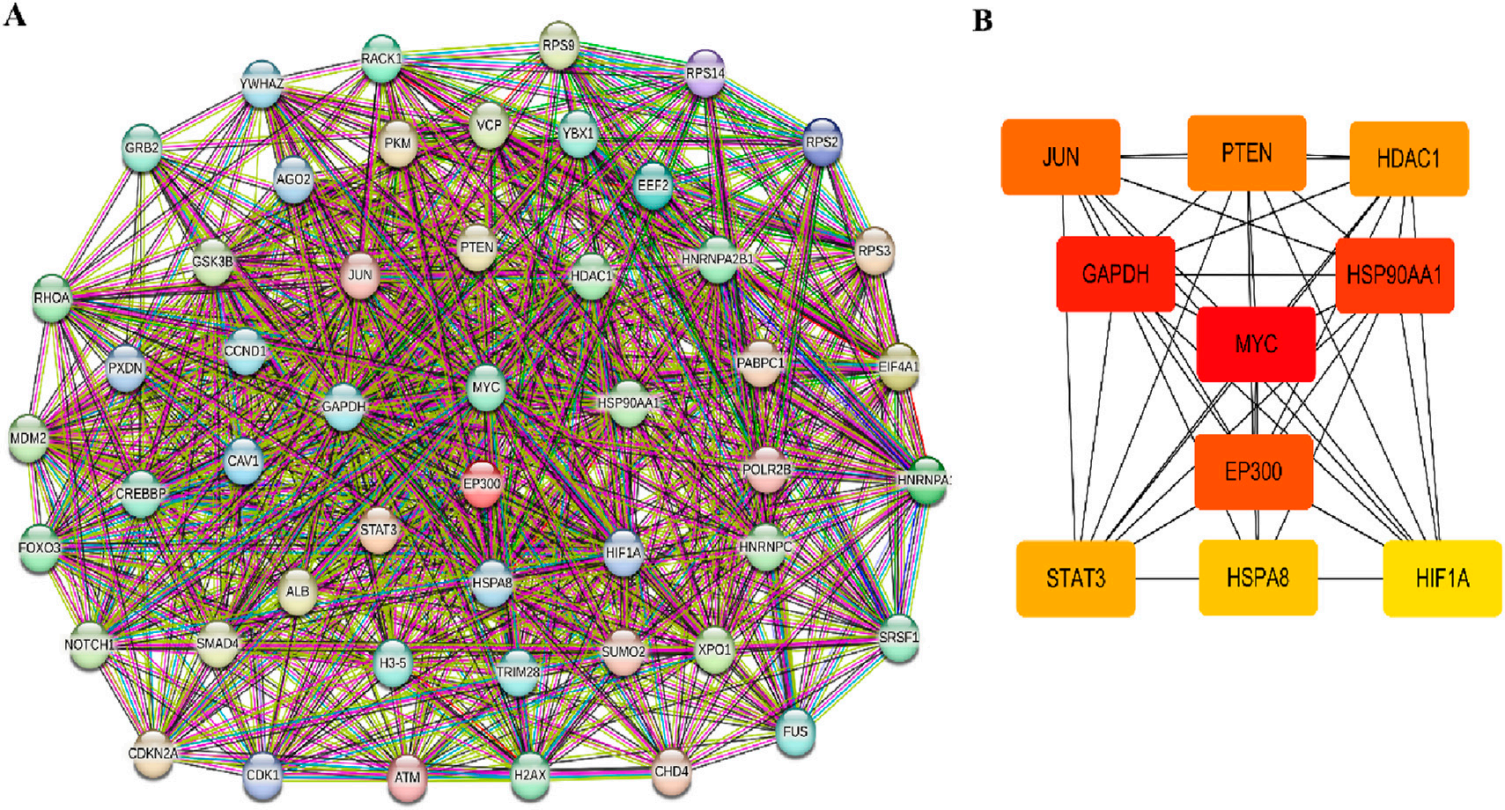
PPI network and hub genes analysis. **(A)** The protein-protein interaction (PPI) network. **(B)** Top 10 hub genes cytoHubba screened in the PPI network.

To investigate the interactive relationships among the identified target genes, a protein-protein interaction analysis was conducted using the STRING database. Focusing on the top 50 target genes, a network analysis based on degree revealed significant interconnectivity among these genes. With a default interaction score cutoff of >0.4, the resulting network consisted of 50 nodes and 700 edges (Figure 4A). Further investigation within the Cytoscape software, utilizing the cytoHubba plugin, pinpointed ten hub genes characterized by the highest network centrality: MYC, GAPDH, HSP90AA1, EP300, JUN, PTEN, DAC1, STAT3, HSPA8, and HIF1A (Figure 4B). Detailed information regarding these hub genes is provided in Table 3.

**TABLE 3:** Top 10 hub genes in network and their functions.

Rank	Name	Score	Functions
1	MYC	288	A proto-oncogene and encodes a nuclear phosphoprotein that plays a role in cell cycle progression, apoptosis and cellular transformation
2	GAPDH	255	Encodes a member of the glyceraldehyde-3-phosphate dehydrogenase protein family. The product of this gene catalyzes an important energy-yielding step in carbohydrate metabolism, the reversible oxidative phosphorylation of glyceraldehyde-3-phosphate in the presence of inorganic phosphate and nicotinamide adenine dinucleotide
3	HSP90AA1	208	The protein encoded by this gene is an inducible molecular chaperone that functions as a homodimer. The encoded protein aids in the proper folding of specific target proteins by use of an ATPase activity that is modulated by co-chaperones
4	EP300	183	Encodes the adenovirus E1A-associated cellular p300 transcriptional co-activator protein. It functions as histone acetyltransferase that regulates transcription via chromatin remodeling and is important in the processes of cell proliferation and differentiation
5	JUN	177	Encodes a protein which is highly similar to the viral protein, and which interacts directly with specific target DNA sequences to regulate gene expression
6	PTEN	169	This gene was identified as a tumor suppressor which negatively regulates intracellular levels of phosphatidylinositol-3,4,5-trisphosphate in cells and negatively regulating AKT/PKB signaling pathway
7	HDAC1	162	The protein encoded by this gene belongs to the histone deacetylase/acuc/apha family and is a component of the histone deacetylase complex. It also interacts with retinoblastoma tumor-suppressor protein and this complex is a key element in the control of cell proliferation and differentiation
8	STAT3	151	The protein encoded by this gene is a member of the STAT protein family. This protein is activated through phosphorylation in response to various cytokines and growth factors. This protein mediates the expression of a variety of genes in response to cell stimuli, and thus plays a key role in many cellular processes such as cell growth and apoptosis
9	HSPA8	146	Encodes a member of the heat shock protein 70 family. It functions as a chaperone, and binds to nascent polypeptides to facilitate correct folding. It also functions as an ATPase in the disassembly of clathrin-coated vesicles during transport of membrane components through the cell
10	HIF1A	143	Encodes the alpha subunit of transcription factor hypoxia-inducible factor-1 (HIF-1). And functions as a master regulator of cellular and systemic homeostatic response to hypoxia, thus plays an essential role in embryonic vascularization, tumor angiogenesis and pathophysiology of ischemic disease

### 3.5 Validation of the DEmiRNAs by qRT-PCR

**FIGURE 5:**
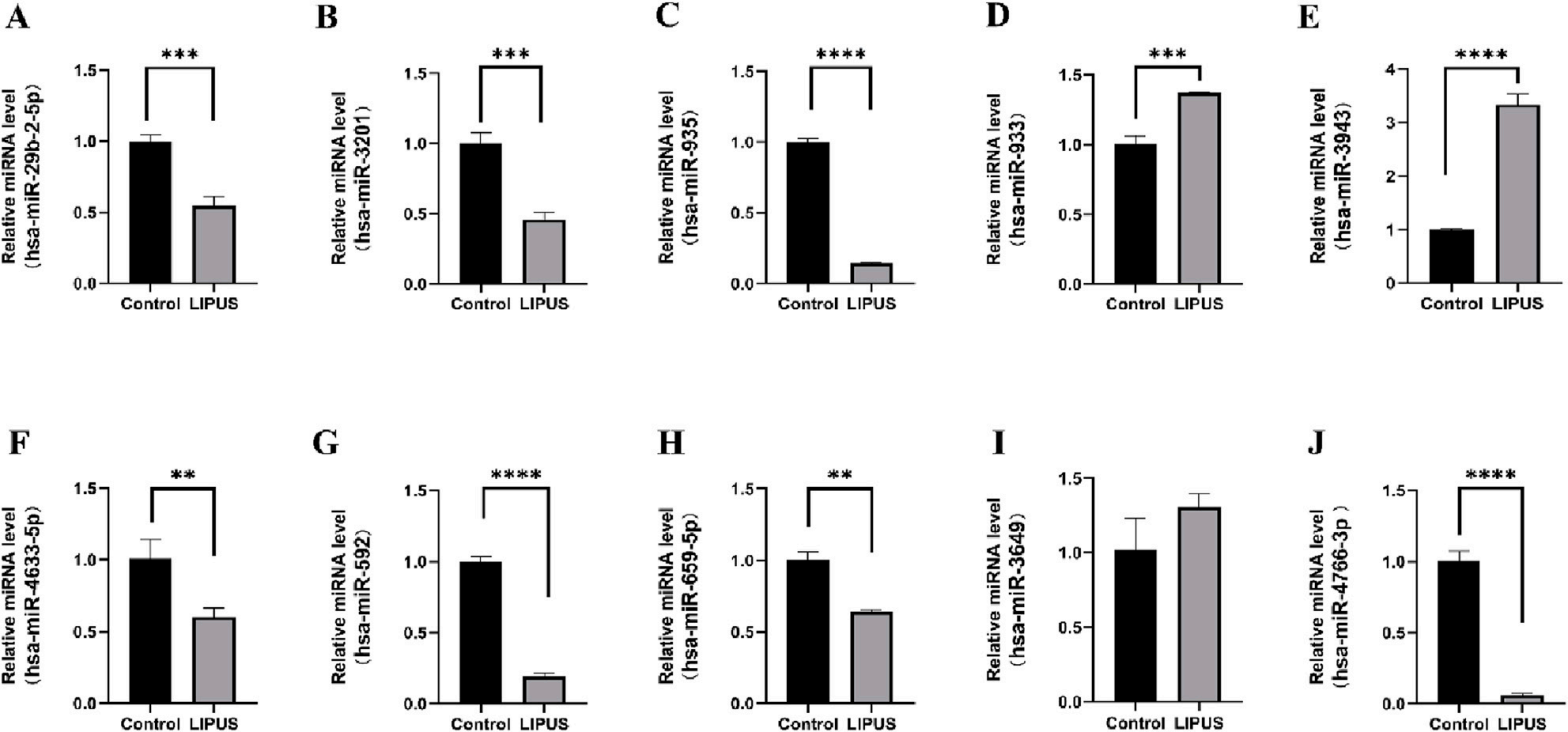
The relative expression of differentially expressed miRNAs. **(A)** hsa-miR-29b-2-5p. **(B)** hsa-miR-3201. **(C)** hsa-miR-935. **(D)** hsa-miR-933. **(E)** hsa-miR-3943. **(F)** hsa-miR-4633–5p. **(G)** hsa-miR-592. **(H)** hsa-miR-659–5p. **(I)** hsa-miR-3649. **(J)** hsa-miR-4766–3p. *p < 0.05, **p < 0.01, ***p < 0.001, ****p < 0.0001.

To validate the bioinformatic predictions of DEmiRNAs, their expression levels were quantified in hUC-MSCs treated with LIPUS. The LIPUS group received 20 min of stimulation at 500 mW/cm^2^. Following treatment, cell supernatants were collected, and EVs were isolated via differential ultracentrifugation. RT-qPCR analysis confirmed the upregulation of hsa-miR-933 and hsa-miR-3943, and the downregulation of hsa-miR-4633-5p, hsa-miR-592, hsa-miR-659-5p, and hsa-miR-4766-3p, which aligned with the bioinformatic results. However, no significant change was observed for hsa-miR-3649. Interestingly, hsa-miR-29b-2-5p, hsa-miR-3201, and hsa-miR-935 displayed upregulation in the GSE188347 dataset but downregulation in our experiments (Figure 5).

## 4 Discussion

SC-EVs possess numerous advantages in the field of regenerative medicine; however, enhancing both the efficacy and yield of SC-EVs is imperative for their clinical translation. Pre-treatment of EV-donor cells presents a promising strategy for manipulating the quantity and composition of EVs. This approach allows researchers to intentionally influence the cargo within EVs, potentially modulating their therapeutic potential for diverse applications. Numerous studies have highlighted that diverse pre-treatment modalities for SCs, encompassing drug administration (Ding et al., 2019; Liu et al., 2020; Yu et al., 2020), hypoxia induction (Chen et al., 2022; Ge et al., 2021; Yuan et al., 2021; Cui et al., 2018), and manipulation of culture media components (Qiu et al., 2020), have the potential to enhance the therapeutic efficacy of SC-EVs. Furthermore, physical stimulation can exert a significant influence on the quantity and composition of EVs (Erwin et al., 2023). LIPUS is a non-invasive and convenient method of physical stimulation. It has demonstrated therapeutic efficacy in treating a variety of conditions, such as delayed wound healing, fracture recovery, osteoarthritis, chronic pain, tendonitis, erectile dysfunction, limb ischemia, dental repair, and central nervous system disorders (Barzelai et al., 2006; Ramli et al., 2009; Martinez de Albornoz et al., 2011; Tanaka et al., 2015; Lin et al., 2016; Clavijo et al., 2017; Poolman et al., 2017; Tanaka et al., 2020). Pre-processing of cells with LIPUS has been shown to effectively modulate both the composition and yield of the EVs they secrete (Li et al., 2019; Zeng et al., 2019; Deng et al., 2021; Liao et al., 2021). These findings collectively paint a promising picture of LIPUS as a potential tool for fine-tuning EV secretion and harnessing their therapeutic potential for diverse applications. The LIPUS dose used in our study (500 mW/cm^2^, 10 min) differed from that employed in the bioinformatic analysis (GSE188347; 90 mW/cm^2^, 30 min). The selection of this latter dose was grounded in our prior research, which established that such a LIPUS dose was capable of enhancing both the yield and the therapeutic effectiveness of SC-EVs. While this discrepancy could potentially influence the observed results, further investigation is needed to fully elucidate the impact of varying LIPUS parameters on SC-EVs.

This study investigated the hypothesis that specific miRNAs and their target mRNAs contribute to the enhanced therapeutic efficacy of SC-EVs treated with LIPUS. We focused on identifying these crucial miRNAs and mRNAs, with the aim of elucidating the mechanisms underlying LIPUS-mediated improvement in SC-EV therapeutic potential. To this end, firstly, we analyzed the GSE188347 dataset through bioinformatics, selecting 10 DEmiRNAs: six upregulated (hsa-miR-3649, hsa-miR-29b-2-5p, hsa-miR-3201, hsa-miR-935, hsa-miR-933, hsa-miR-3943) and 4 downregulated (hsa-miR-4633-5p, hsa-miR-592, hsa-miR-659-5p, hsa-miR-4766-3p). We then constructed a miRNA-mRNA interaction network to uncover target genes of these DEmiRNAs. Subsequent functional and pathway enrichment analyses of these targets explored their potential roles. To identify key regulatory hubs, we constructed a PPI network and utilized the Cytoscape plugin CytoHubba. Finally, qRT-PCR validated the expression of these DEmiRNAs.

MicroRNAs are small, non-coding RNAs that regulate gene expression by binding to mRNAs and promoting their degradation or translation inhibition (Gulyaeva and Kushlinskiy, 2016). These versatile molecules play crucial roles in diverse biological processes, making them attractive targets for therapeutic intervention in various diseases. Among the 10 DEmiRNAs potentially enhancing the therapeutic efficacy of SC-EVs, many have shown promise as therapeutic agents across diverse pathologies. For instance, miR-29b-2-5p suppresses cell proliferation, induces cell cycle arrest, and promotes apoptosis in pancreatic ductal adenocarcinoma by targeting Cbl-b, thereby enhancing p53 expression (Li et al., 2018). Although the function of miR-3201 remains controversial, its downregulation in recurrent epithelial ovarian cancer suggests a tumor-suppressive role during cancer recurrence (Chong et al., 2015). However, other studies have implicated miR-3201 in several cancer-promoting pathways, highlighting the need for further investigation (Su et al., 2018). miR-935 exhibits a protective role against oxidative stress in cardiac progenitor cells and inhibits the proliferation and invasiveness of glioma cells, suggesting its potential as a therapeutic agent in both cardiac and cancer settings (Zhang et al., 2021; Aguilar et al., 2023). miR-933 might control hyperglycemia and hyperinsulinism by regulating ATF2 target genes, potentially playing a role in type II diabetes mellitus pathogenesis (Islam et al., 2020). miR-4633–5p serves as a potential biomarker and tumor suppressor in metastatic melanoma (Zou et al., 2018), while miR-592 exhibits diverse roles in various cancers, including hepatocellular carcinoma and breast cancer (Jia et al., 2016; Hou et al., 2017). miR-659–5p, regulated by hsa_circ_0000911, is an emerging target of the MAPK pathway in breast cancer (Liu et al., 2022). While limited information is currently available on the therapeutic potential of miR-3649 and miR-4766–3p, ongoing research extensively investigates their roles in various diseases and their potential as therapeutic targets.

This study acknowledges certain limitations. Firstly, the initial study (GSE188347) utilized stem cells from the apical papilla as the source of EVs. However, to validate the expression levels of DEmiRNAs, we opted for hUC-MSCs due to their wider usage in medical research. This methodological difference may introduce some bias when analyzing the DEmiRNAs. The discrepancy between the bioinformatics data (from the GSE188347 dataset) and our real validation data might be attributed to the different cell types used. Although both are stem cells, they may have inherent differences in their gene expression profiles and responses to LIPUS treatment, which could potentially lead to variations in the expression levels of miRNAs. Further studies are needed to fully understand the impact of cell type on the miRNA expression and the therapeutic potential of LIPUS-treated SC-EVs. Furthermore, the intricate nature of gene function and underlying molecular mechanisms necessitates further investigation through cellular and animal research models.

## 5 Conclusion

In summary, this study identified differentially expressed miRNAs and mRNAs associated with potential therapeutic roles in low-intensity pulsed ultrasound treated stem cell-derived extracellular vesicles. These findings suggest novel therapeutic targets for LIPUS and hold promise for clinical applications. Future studies will focus on conducting *in vitro* and *in vivo* functional assays to further validate and elucidate the biological roles of these identified mRNAs.

## Data Availability

Publicly available datasets were analyzed in this study. This data can be found here: GEO Accession: GSE188347 (https://www.ncbi.nlm.nih.gov/geo/query/acc.cgi?acc=gse188347).

## Abbreviations

LIPUS, low-intensity pulsed ultrasound; EVs, extracellular vesicles; hUC-MSC, human umbilical cord mesenchymal stem cells; SCs, stem cells; miRNAs, microRNAs; SC-EVs, stem cells-derived EVs; DEmiRNAs, differentially expressed miRNAs; PCA, Principal component analysis; KEGG, Kyoto Encyclopedia of Genes and Genomes; BP, biological process; GO, Gene Ontology; CC, cellular component; MF, molecular function; PPI, protein-protein interaction.
